# Intention‐to‐treat analyses and missing outcome data: A tutorial

**DOI:** 10.1002/cesm.12075

**Published:** 2024-05-21

**Authors:** Marty Chaplin, Kerry Dwan

**Affiliations:** ^1^ Cochrane Infectious Diseases Group Liverpool School of Tropical Medicine Liverpool UK

## Abstract

This tutorial focuses on “intention‐to‐treat” analyses and missing outcome data in systematic reviews. There is a lack of consensus on the definition of the ITT approach. We will explain the principles of an intention‐to‐treat analysis, and outline the key issues you need to consider when planning, conducting and writing up your systematic review.

ITT micro learning module

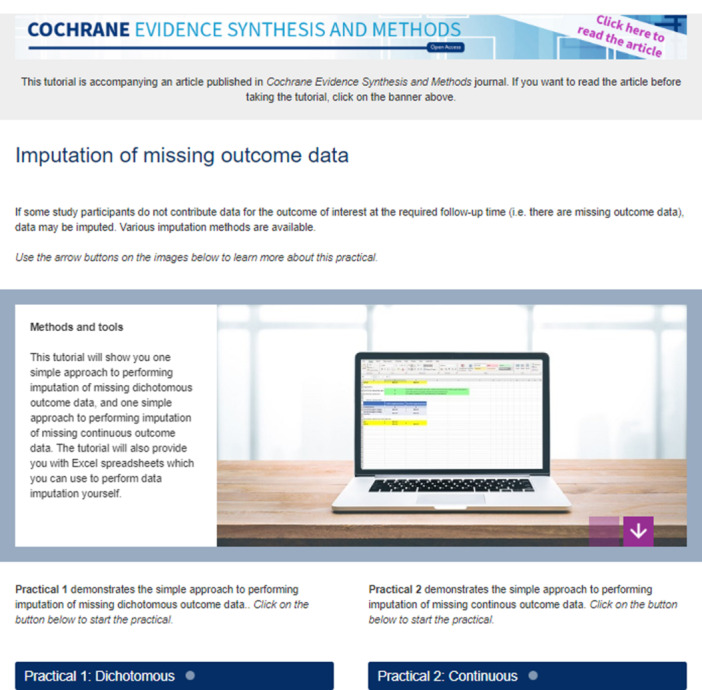

This tutorial focuses on “intention‐to‐treat” analyses and missing outcome data in systematic reviews. There is a lack of consensus on the definition of the ITT approach. We will explain the principles of an intention‐to‐treat analysis, and outline the key issues you need to consider when planning, conducting and writing up your systematic review.

## 1 WHAT IS AN INTENTION‐TO‐TREAT ANALYSIS?

The authors of studies included in systematic reviews may use the term “intention‐to‐treat” or “intent‐to‐treat” (ITT) to describe the approach taken when reporting and analyzing outcome data. The ITT approach has two principles.

*Principle A: Outcome data are reported and/or analysed according to the participant's assigned intervention, regardless of the intervention they actually received or their adherence to their assigned intervention. For randomised controlled trials, this approach is sometimes referred to as an “as‐randomised” analysis*.

This principle is not met if study authors:

(i) Report and/or analyze outcome data for participants according to the intervention they actually received (this approach is sometimes referred to as an “as‐treated analysis”).
(ii) Report and/or analyze outcome data only for participants who adhered sufficiently to their assigned intervention (this approach is sometimes referred to as a “per‐protocol analysis”).

Study authors make decisions about which approach to take based on whether they are interested in determining the effect of allocation to an intervention (regardless of whether the intervention was received as intended), the effect of receiving an intervention, or the effect of adhering to an intervention (as specified in the trial protocol).

*Principle B: Outcome data are measured for all randomised participants*.

If some participants do not contribute data for the outcome of interest at the required follow‐up time (i.e., there are missing outcome data), data may be imputed. Various imputation methods are available, from simply assuming that all participants with missing data had a particular outcome (e.g., study authors may assume that all participants with missing data experienced a poor outcome, such as treatment failure), to more complex methods such as multiple imputation.

This principle is not met if study authors report and/or analyze outcome data only for participants with nonmissing outcome data (this approach is sometimes referred to as a “complete‐case analysis”).

When choosing whether to ignore or impute missing data, and when selecting an imputation method, study authors should consider whether missing data are likely to be “missing at random” or not. Data are “missing at random” if the fact that the data are missing is unrelated to the true data values. Complete‐case analyses, and some imputation methods, may lead to biased results if the missing data is “missing not at random.” Table 1 provides examples of data that are “missing at random” and data that are “missing not at random.”

**Table 1:** Examples of data that are “missing at random” and “missing not at random.”

	Example
Missing at random	In a study of different interventions for treating anemia in pregnant women, if blood samples for some individuals were damaged in the laboratory due to human error, hemoglobin level measurements for these individuals are **missing at random**.
Missing not at random	In a study of different interventions for treating migraines, if individuals withdraw from the study as they do not believe the intervention they are receiving is having any impact on their migraines, and therefore do not attend their final follow‐up visit, data for these individuals are **missing not at random**.

There is no consensus on the definition of the ITT approach [1, 2]. Some study authors use the term ITT when applying both principles; others use the term when applying just one principle. Study authors may use the term “modified ITT” approach, which also has no consistent definition. The estimated intervention effect in a study may be impacted by the study author's choice of ITT approach. If this study is then included in a systematic review, pooled results and review conclusions may also be impacted. Ideally, when study authors refer to an ITT or modified ITT approach, they clearly specify the intended meanings of these terms. However, this is unfortunately not always the case.

## 2 WHAT DO I NEED TO THINK ABOUT WHEN…

- *Writing my protocol?*

If you use the term “ITT” in your protocol, clearly define what you mean by this term.

First, specify whether you are interested in determining the effect of allocation or adherence to a particular intervention. If you are interested in determining the effect of allocation to an intervention, then you should state that, wherever possible, you intend to extract outcome data that are reported and/or analyzed according to the participant's assigned intervention, regardless of the intervention they actually received or their adherence to the intervention. If instead, you are interested in determining the effect of adherence to an intervention, state that you intend to extract outcome data from analyses that estimate per‐protocol effects (see Cochrane Handbook [1] Section 8.2.2 for discussion of different approaches to estimation of per‐protocol effects, and the biases associated with these approaches).

Review authors should be aware that data from the preferred analysis set may be unavailable, and it may be necessary to extract data from an alternative analysis set, if these are the only data available. If this is the case, you should decide whether to:

(i) include the data in your analyses (and conduct sensitivity analyses excluding this data from your analyses);
(ii) exclude the data from quantitative analyses, and present the data in tables or narratively, instead.

The appropriate approach is likely to depend on whether the number of participants included in the alternative analysis set differs from the number of participants who would have been included in your preferred analysis set to such an extent that there could be a substantial impact on the study results.

Second, specify how you will handle missing outcome data in your review. You may choose to:

(i) include data from analyses in which study authors have performed imputations of missing data (if available);
(ii) impute missing data yourself; various imputation methods are available (see Cochrane Handbook [1] Chapters 10.12.2 and 10.12.3);
(iii) include data from a complete‐case analysis (if available).

Sensitivity analyses can be performed to investigate the impact of your chosen approach. The appropriate approach is likely to depend on various factors, including the extent of missing data, the study authors' imputation method (if applicable), and whether complete‐case analysis results (if available) are likely to be at high risk of bias due to missing outcome data.

Although it is important to consider the issues outlined above when writing the protocol, it may be appropriate to make some decisions regarding your methods at a later stage, once you have identified studies, extracted data and assessed risk of bias. You can outline and justify your chosen methods when completing the review write‐up.

- *Extracting data?*

Carefully examine the publications of included studies (including the statistical methods section, participant flow diagrams, and results tables) to determine which, if any, ITT principles were adopted for the analyses. If the approach is not clear from information provided in the paper, you can contact the study authors. If you unable to clarify the approach with the authors, you should decide whether to include the data in your analyses (and conduct sensitivity analyses exploring the impact of including the data on your results), or to exclude the data from analyses (and present the data in tables or narratively, instead).

- *Assessing risk of bias*

ROB2 [3] (for RCTs) and ROBINS‐I [4] (for nonrandomised studies of interventions) assessments for one domain (“Bias due to deviations from intended interventions”) vary according to whether you are interested in determining the effect of allocation or adherence to the intervention (as specified in your review protocol). These tools also address risk of bias due to missing outcome data. It is highly likely that answering the signaling questions in ROB2 and ROBINS‐I will guide your decisions about the appropriate analysis approach to take in your review. Detailed guidance on both tools is available (www.riskofbias.info).

- *Writing up my review?*

If you use the term “ITT” in your review, clearly define what you mean by this term. It should be clear to the reader whether data from included studies were analyzed using an “as‐randomised” or an “as‐treated/per‐protocol” approach, and how missing outcome data were handled. If you imputed any missing data yourself, identify the studies you performed imputations for and provide details of the imputation approach used.

It is important that systematic review methods are explicit and justified. If you made decisions during the review process about whether to include or exclude data from analyses, or to perform imputations, provide the rationale for these decisions. The methods section, results section and analysis footnotes can all be used to ensure the transparency of your approach. You should also outline any updates to the methods specified in the protocol under the “Differences between protocol and review” heading.

## 3 FURTHER READING AND ONLINE CONTENT

Professors Altman and Bland discuss missing data as part of the BMJ “Statistics Notes” series [5].

The Cochrane Handbook for Systematic Reviews of Interventions [1] provides information on intention‐to‐treat analyses and missing outcome data in Chapters 8.2, 8.4, 8.5, and 10.12, including five general recommendations for dealing with missing data in Cochrane Reviews.

**Figure 1:**
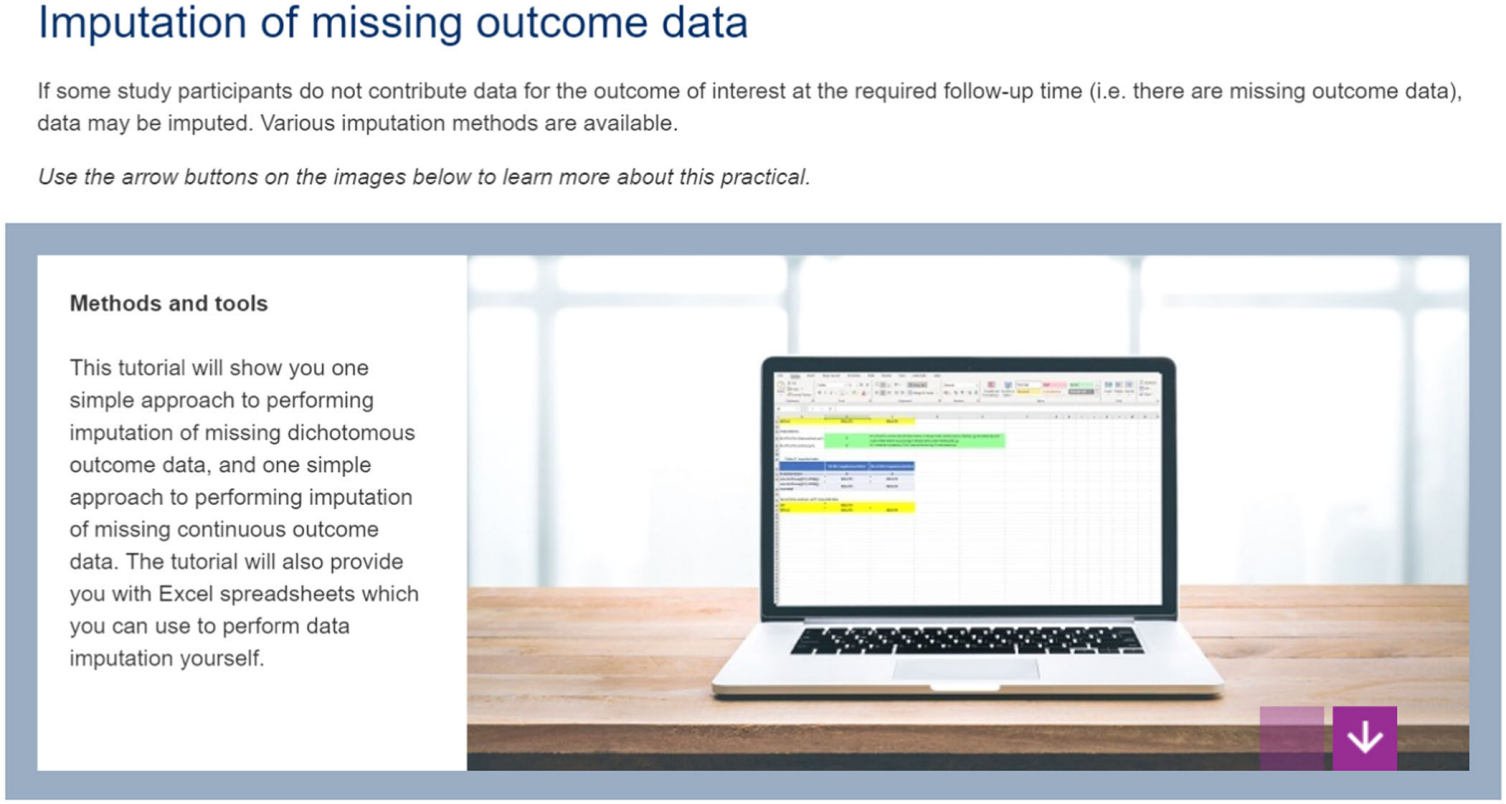
Screenshot of micro learning module.

Cochrane Training have produced a micro learning module [6] demonstrating one simple approach to imputation of missing dichotomous outcome data [7] and one simple approach to imputation of missing continuous outcome data [8] (Figure 1).

## AUTHOR CONTRIBUTIONS

**Marty Chaplin**: Conceptualization; writing—original draft; writing—review and editing. **Kerry Dwan**: Conceptualization; supervision; writing—review and editing.

## CONFLICT OF INTEREST STATEMENT

The authors declare no conflict of interest.

## PEER REVIEW

The peer review history for this article is available at https://www.webofscience.com/api/gateway/wos/peer-review/10.1002/cesm.12075.

## Data Availability

Data sharing not applicable – no new data generated.
